# Microscopic Examination of the Contents of the Hepatic Ducts, with Conclusions Founded Thereon

**Published:** 1849-12

**Authors:** T. Wharton Jones


*Microscopic Examination of the Contents of the Hepatic Ducts, with Conclusions founded thereon.* By T. Wharton Jones, F. R. S.—In the contents of the larger hepatic ducts there are seen, on microscopical examination: 1st, the detached columnar epithelium cells of the ducts themselves; 2d, free oval nuclei, which appear to have been derived from cells of this kind; 3d, free round nuclei, apparantly derived from the proper hepatic cells; 4th, minute granules, free or in amorphous flakes, globules of oil, and fragments of cell walls. In addition to these, in the smaller ducts, are found cells resembling those of the parenchyma of the liver, except in being usually paler, on account of the contained granules and globules being fewer and more minute. Having, by way of comparison, examined the contents of the pancreatic duct, and recognized in it, with its own columnar epithelium, the free nuclei and granular amorphous matter resulting from the disintegration of the secreting cells developed within vesicles of that gland, the author considers that, from the analogy of the products of the secreting action in the two cases, an argument may be fairly drawn in favor of the analogy in essential nature between the cells of the hepatic parenchyma and the endogenous cells of the pancreas and other glands of ordinary construction. This deduction helps to confirm the view of the structure of the liver propounded by Dr. Leidy.—*Phil. Trans.*, 1848.

